# Is there a contextual interference effect for sub-elite alpine ski racers learning complex skills?

**DOI:** 10.3389/fbioe.2022.966041

**Published:** 2022-09-15

**Authors:** Christian Magelssen, Per Haugen, Robert Reid, Matthias Gilgien

**Affiliations:** ^1^ Institute for Physical Performance, Norwegian School of Sport Sciences, Oslo, Norway; ^2^ The Norwegian Ski Federation, Oslo, Norway; ^3^ Center of Alpine Sports Biomechanics, Engadin Health and Innovation Foundation, Samedan, Switzerland

**Keywords:** contextual interference, motor learning, alpine ski racing, sport expertise, retention, pumping to increase velocity, course setting

## Abstract

Scientific understanding of the contextual interference effect stems mainly from studies on unskilled participants learning artificial laboratory tasks. Although one goal of such studies is to extrapolate the findings to include real-world learning situations such as sports, this generalization is not straightforward. This study tested the contextual interference effect with 66 sub-elite, competitive alpine ski racers who learned a new movement pattern−the pumping technique to increase velocity in slalom−by practicing this skill in three different slalom courses over a 3-day training period. The interleaved group practiced all three courses each day in a semi-random order. In contrast, the blocked group practiced only one course each day, which was randomized and counterbalanced across the participants in this group. A retention test was delivered 72 h after the last practice day. In contrast to our hypothesis, the interleaved group did not display significantly better retention than the blocked group. The interleaved group’s performance was also not significantly attenuated during skill learning compared to the blocked group. Our results underscore the importance of conducting motor learning experiments in natural environments to understand the conditions that facilitate learning beyond the laboratory environment.

## 1 Introduction

Many studies suggest that training with a high degree of contextual interference can create favorable conditions for learning motor skills (Magill and Hall, 1990; Lee and Simon, 2004; Merbah and Meulemans, 2011). Experiments on the contextual interference effect usually introduce learners to three tasks to be learned (tasks A, B, and C). In the high contextual interference condition (i.e., interleaved practice), the practice order of tasks makes the learner frequently switch between the tasks they acquire (for example, ABC, BAC, or CBA). In contrast, less switching occurs in the low contextual interference condition (i.e., blocked practice) by arranging the tasks in blocks (for example, AAA, CCC, BBB). Previous research has provided evidence that interleaved practice often improves skill preservation over time (i.e., retention) and adaptation of the skill to new situations (i.e., transfer) compared to blocked practice. However, a notable aspect is that blocked practice often results in superior performance during skill acquisition compared to the interleaved group. This paradoxical interaction—called the contextual interference effect—represents a prime example of the distinction between performance and learning in motor learning and has been extensively replicated in a wide variety of scientific laboratory experiments (Shea and Morgan, 1979; Lee and Magill, 1983; Simon and Bjork, 2001; Thomas et al., 2021).

Despite the existence of ample evidence for the contextual interference effect being present in laboratory environments, it has become clear that the principles deriving from the research do not always generalize to the learning of motor skills in naturalistic settings such as sports (Wulf and Shea, 2002; Brady, 2004; Barreiros et al., 2007). For example, Barreiros et al. (2007) have reported that the proportion of studies showing improved retention due to interleaved practice was considerably smaller for skills performed in a natural environment than for skills performed in a laboratory environment. Furthermore, a meta-analysis showed that the contextual interference effect is typically smaller and more dispersed than in laboratory tasks (Brady, 2004). Therefore, while interleaved practices may improve learning for simple tasks, the evidence for contextual interference for learning more complex tasks in natural environments is not conclusive.

Over the years, researchers have proposed and examined several different moderators to account for the contradictory results between laboratory and natural environments, including the learner’s age (Del Rey et al., 1983), the amount of practice (Shea et al., 1990), the type of task (Magill and Hall, 1990), the modality-specific requirements of the task (Schöllhorn et al., 2022), and the learner’s skill level relative to the difficulty of the task (Wulf and Shea, 2002; Guadagnoli and Lee, 2004). Concerning the latter of these moderators, the challenge-point framework (Guadagnoli and Lee, 2004) posits that the efficacy of interleaved practice depends on the difficulty of the task as it is objectively defined (that is, nominal task difficulty) but also how challenging the task is relative to the learner’s skill level and practice environment (that is, functional task difficulty). The framework predicts that an interleaved practice may be more beneficial to promoting learning in a context involving learning a task with low nominal difficulty (for example, a simple laboratory task). This expected observation is because interleaved practice increases the functional difficulty of the task to engage the cognitive mechanisms responsible for causing the contextual effect. With more nominally difficult tasks, the task’s characteristic may already be sufficiently challenging to achieve this end so that beginners may benefit from the blocked practice. However, as learners become better at the task, increasing the functional difficulty of the task through interleaved practice may be needed to engage the cognitive mechanisms to promote additive learning. In support of the challenge-point framework, several studies have provided evidence that providing beginners with a gradual and systematic increase in contextual interference when learning complex skills seem to be a better learning approach than the sole use of blocked or interleaved practice (Porter and Magill, 2010; Saemi et al., 2012). These findings suggest that the optimal practice condition changes with the learner’s proficiency and the skill’s complexity.

The challenge point framework and the supporting evidence that the learner’s skill level interacts with the characteristic of the task in determining the contextual interference effect can build the impression that skilled performers benefit from training with a high degree of contextual interference when improving or refining their skills. Even though some researchers have advocated such an approach (Christina and Bjork, 1991; Schmidt, 1991), few studies have explicitly tested it. One of the few exceptions is a study on skilled baseball players who performed additional batting training to probe the contextual interference effect (Hall et al., 1994). Three groups practiced batting with three types of baseball pitches. The blocked group practiced these pitches in a blocked order (AAA, BBB, CCC), whereas the interleaved group practiced them in a random order (BCA, ABC, BAC). At the end of the training intervention, the interleaved group performed better than the blocked group. This study demonstrated that interleaved practice might also improve learning for skilled performers. It is important to note that Hall et al. (1994) used variations of a single skill (i.e., batting) to probe the contextual interference effect. In a recent study, Buszard et al. (2017) performed a between-skill manipulation to examine the contextual interference effect in youth tennis players. While interleaving the practice schedule did not improve retention for these players compared to the blocked practice schedule on the same task, there was evidence that the interleaved group transferred their skill better to competition (i.e., transfer). Hence, it remains unclear whether training with contextual interference improves learning for skilled performers when improving their skills. This lack of understanding is critical to address in order to provide proper recommendations for instructors in sports and other motor activities, such as surgical operations in medicine and the training of military personnel.

Testing the contextual interference effect on skilled performers implies specific challenges that must be overcome and effectively solved. The biggest challenge is that skilled performers are usually obsessed with achieving success in their activity and devote significant amounts of their time and resources to improving their performance in this activity. Therefore, recruiting them for a study is often challenging because of their reluctance to modify their training for an experiment, especially if it does not lead to immediate performance gains (Farrow and Buszard, 2017). Even if performers were willing to participate, it would often require a large volume of practice to improve the performance of a skilled practitioner compared to a novice performer (Hall et al., 1994). Hence, even if interleaved practice makes the training more effective, the effect may only become visible after extensive practice, regardless of the skills training method. A final obstacle is that it is often difficult to achieve a robust and sensitive performance goal, especially in alpine ski racing, where external conditions such as snow and wind vary considerably and may influence performance (Williams et al., 2017). Overcoming these challenges requires in-depth knowledge of the skill domain, and real-world practitioner skills are needed to invent innovative approaches to assess skills and deal with issues of validity at the same time (Farrow and Buszard, 2017).

Considering the need for a better understanding of how the contextual interference effect translates to skilled learners, and how to cope with the described challenges, we have investigated the contextual interference effect on skilled athletes in the complex sport of alpine ski racing in this study. Alpine ski racing is a sport where performance is measured as the time from start to finish, where athletes need to pass through a pre-defined course marked with gates. The sport consists of six main disciplines: slalom (SL), giant-slalom (GS), super-G (SG), downhill (DH), Parallel and Combined, which vary in the number of direction changes,timing and dynamics in turns,terrain and transitions, course length, and jumps (Gilgien et al., 2018). Of these six disciplines, slalom skiing is the most technically demanding due to its frequent changes of direction, high turn forces, and small turn radii (Reid, 2010). Slalom courses generally comprise ∼50 gates, adjacently positioned with a linear distance of 6–13 m. These courses can vary extensively between races depending on the course setter, usually a coach who can determine the type of course within the rules of Fédération International de Ski (FIS). Besides the variability in courses, there is also large variability in terrain characteristics (for example, incline and terrain transitions), snow properties, and weather (for example, visibility). Slalom racers should therefore expect a significant degree of variability in conditions in the performance arena.

Although the total time differences between skiers in slalom races can be quite small, section differences through a course can be quite significant while typically equalizing to small differences at the finish (Supej and Cernigoj, 2006; Supej and Holmberg, 2011). The sections of slalom courses where significant time differences typically occur between skiers are flat terrain sections (Supej and Cernigoj, 2006). An essential characteristic of flat sections is that the component of gravity that accelerates the skiers downhill is small (Reid, 2010). Therefore, the skier must make the necessary adjustments to their technique to ski fast in this type of terrain (Supej et al., 2015). One technique proposed to help increase speed in flat terrain is to “pump” while turning to increase the turn exit speed (Mote and Louie, 1983; Lind and Sanders, 2004). In this context, pumping refers to the technique of extending the legs and pushing the center of mass towards the axis of rotation at the center of the turn. Through the conservation of angular momentum, pushing the center of mass closer to the axis of rotation can lead to increased tangential velocity (Lind and Sanders, 2004). Therefore, the extent and quality with which skiers can exploit this technique can be a primary explanation for the time differences in flat sections.

Because the technique that leads to good performance differs depending on the terrain incline, researchers have recommended dividing training into sessions with uniform terrain inclines to achieve more element-focused training (Supej et al., 2015). Once training in a section of uniform terrain incline, coaches need to determine the slalom gates’ location down the hill. The location of the gates determines two characteristics of the course: the linear distance between successive gates determines the room skiers have for turning between gates, and the offset determines how “turny” the course is. Changes in these two course dimensions can cause significant changes in the required technique, and the tactics skiers must use to ski the course. For example, changes in gate offset have been shown to reduce speed and turn radius but increase turn forces, impulse (a measure of physical load), and inward lean for giant slalom and super-G (Spörri et al., 2012; Gilgien et al., 2020, Gilgien et al., 2021). In contrast, shortening the linear distance between gates causes a reduction in turn time and speed but has a limited effect on forces and turn radii compared to changes in the offset (Reid, 2010; Gilgien et al., 2020, 2021). Because course setting has a significant impact on skiers’ technique and is the training variable that coaches can influence the most, there is a general conception that this is one of the most critical variables affecting learning.

Because skiers never know what courses and conditions to expect in a race, they must master an extensive range of conditions. Therefore, undertaking training to perfect performance in a single course setting may not be effective. Instead, researchers have argued that a better approach is to use interleaved practice in these types of open sports (Farrow and Buszard, 2017). However, few studies have tested this recommendation due to the described challenges of conducting studies on complex learning tasks with skilled performers. Therefore, we established this study to test the contextual interference effect with skilled alpine ski racers in a realistic real-world ski racing environment. An important goal was to do the study with a large number of participants to estimate the contextual interference effect robustly. To achieve this goal, we designed a study that targeted a particular skill element of skiing performance that was relevant for the skiers to improve. Targeting this specific element instead of providing holistic training, we were also able to improve the skiers’ performance by a significant degree, because training this skill was novel for the participants.

In this study, we expected contextual interference to apply to the training of alpine ski racers. Our rationale for expecting an extension of the effect to this context emerged from previous studies that reported improved retention of continuous skills (cyclic bimanual coordination task) resulting from interleaved compared to blocked practice (Tsutsui et al., 1998; Pauwels et al., 2014). Moreover, in a snow environment, Smith (2002) reported that novices learned snowboarding turns better after practicing four different turns (left/right and heel/toe) in an interleaved compared to a blocked order. This finding suggests that contextual interference may be relevant for learning skills in alpine ski racing. If skiers vary their turns in an interleaved manner, as is accomplished by frequently switching between courses, we could expect to observe contextual interference in alpine skiing. In the snowboarding study, however, the participants were novices, and it is unclear how this extrapolates to skilled performers. Based on the previous research that has provided evidence for the contextual interference for experienced performers (Hall et al., 1994), we hypothesized that interleaved practice would suppress performance during acquisition but improve performance at retention.

## 2 Material and methods

### 2.1 Participants

Sixty-six competitive alpine ski racers (31 females), aged between 14 and 28 (*Mean age* = 17, *SD* = 2.7 years), were recruited from three different ski clubs and four high school-level development academies affiliated with the Norwegian Ski Federation (NSF). Except for a smaller subset of the participants (*n* = 12) who competed in national races for skiers between 14 and 16 years, the participants had participated in Fédération International de Ski (FIS) races and had recorded FIS points. Our sampling approach was to recruit as many skiers as we had access to in eastern Norway. By the end of the experiment, we had recruited participants from almost every ski academy in the region. Unfortunately, 12 of these 66 participants either ended up in Covid-19 quarantine after the last practice session, or were sick, reducing the sample to 54 participants who completed the entire protocol. Given this final sample size, the study had 11% and 44% power to detect a small and medium-sized effect, respectively, in comparing groups in the main outcome (see Supplementary Material for specific details of the power calculations).

All participants provided written informed consent prior to the study. Where participants were less than 15 years of age, we also required informed consent from their parents/guardians. The protocol was approved by the Human Research Ethics Committee of The Norwegian School of Sport Sciences.

### 2.2 Task and apparatus

Our intervention targeted the improvement of the pumping technique in the discipline of slalom skiing. To create a learning situation where the pumping skill could be trained and assessed under controlled and stable conditions, we used a 250-meter-long, relatively flat section of the race hill in the indoor skiing hall in Oslo, Norway (https://snooslo.no/). Having stable external conditions was especially important in this study because the intervention spanned multiple days, and changes in external conditions could influence the results. Therefore, we performed the study indoors, obtaining stable wind and light conditions. Only minor changes in temperature and snow conditions needed consideration, which we dealt with by water-injecting the snow before each round of data collection to provide race-like snow surface conditions. In addition, the snow surface was maintained manually before and during each day of training and testing. We recorded the temperatures in the snow hall, and we asked participants individually to rate the snow conditions after each ski day.

**FIGURE 1:**
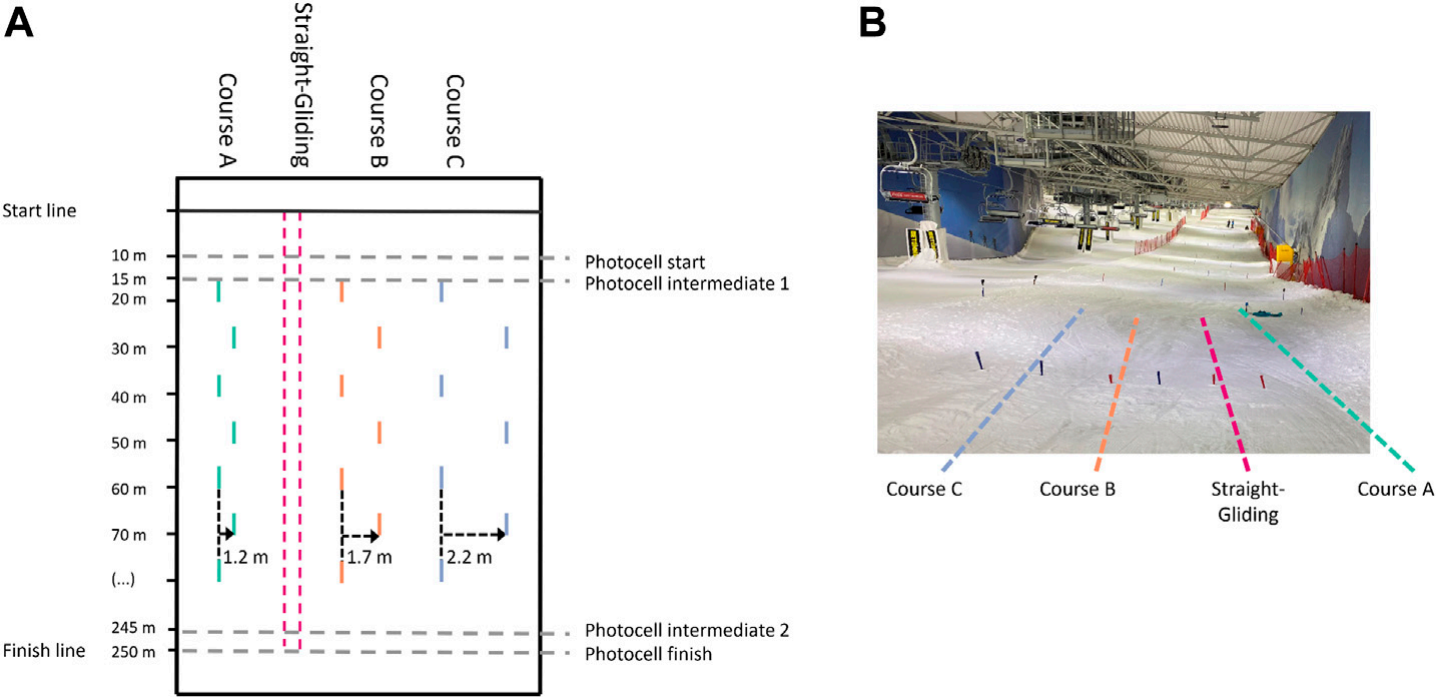
The experimental set-up of this study. **(A)** This figure depicts the three slalom courses with the different offsets. In the straight-gliding line between course A and course B, participants skied the section straight down in a static, upright position. Timing started when the participant crossed “photocell start” and ended when the participant crossed “photocell finish”. **(B)** The same courses seen from the starting area.

To create tasks that required different ways of pumping (that is, different timing and amplitude), we set three slalom courses (A, B, and C) that had different gate offsets: for courses A, B, and C, we used 1.2-, 1.7- and 2.2-meter gate offsets, respectively (Figures 1A, B). However, all courses had a vertical gate distance of 10 m due to space constraints in the skiing hall that forced us to set the courses parallel. We deliberately chose the specific gate offsets from several pilot tests with skiers that fitted the participants’ skill levels in the study. The courses were within the range that let participants pump yet were perceived as very different types of courses. Performance time was measured with photocells set up 10 m after the start and at the finish. The time started when the participant crossed the first photocell and stopped when the participant crossed the last photocell (Figure 1A). We used a wireless photocell timing system (HC Timing wiNode and wiTimer, Oslo, Norway) to measure these times.

Participants performed all runs from the same starting line and with a standardized starting procedure to avoid confounding the task with different entrance speeds into the course. Participants had to start in a stationary position and ski straight out of the starting gate to the first photocell with no poling or skating to generate propulsion.

Besides using the ski hall to limit the impact of external conditions on performance, we included three straight gliding runs each day to capture the effect of any potential change in snow conditions on performance. The straight gliding lane was set between courses A and B, where the participants skied the 250-meter section in a straight line from start to finish. The straight gliding was completed in an upright, stationary posture to create a similar drag area for each run. This procedure allowed us to evaluate changes in ski snow friction that were not influenced by changes in air drag (due to changes in the frontal area). Including the straight gliding runs enabled us to normalize the performance time results between different test days.

### 2.3 Procedure

**FIGURE 2:**
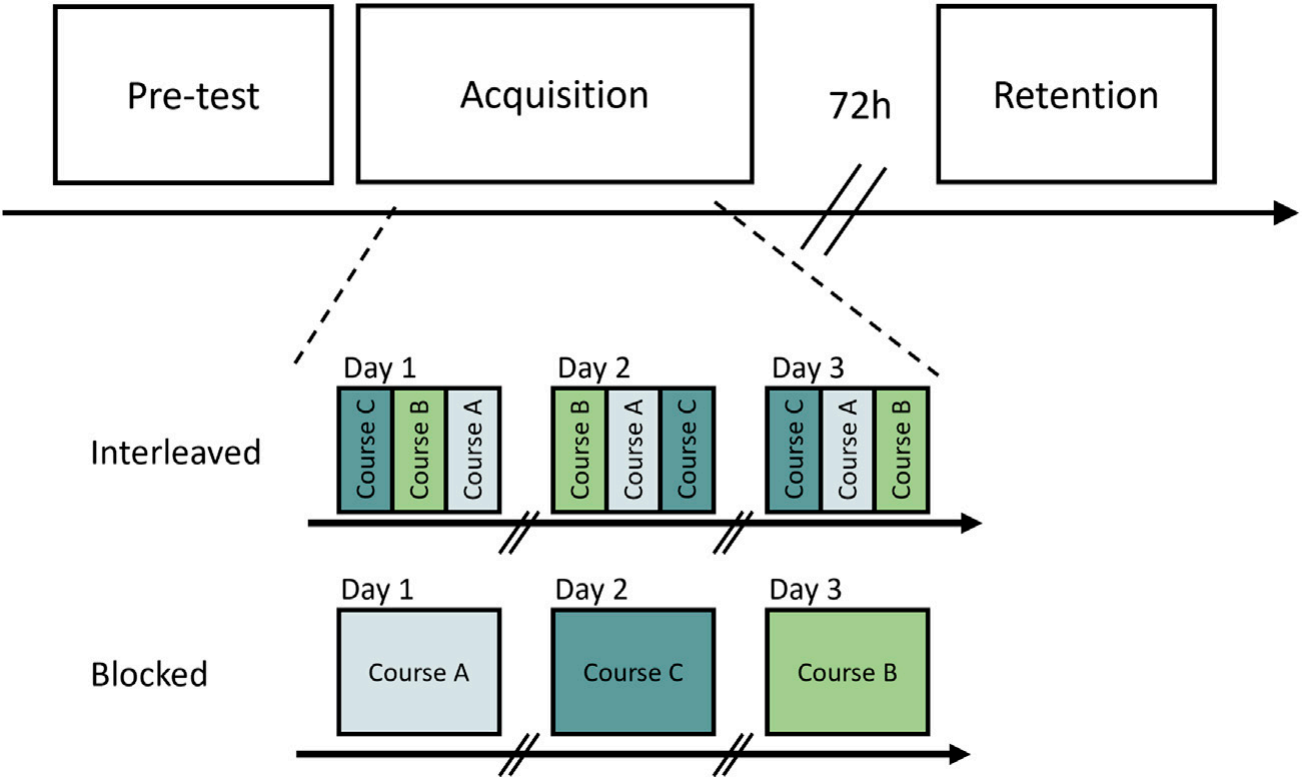
The design of this study. On day 1, all participants performed a pre-test that consisted of three runs in each of the three slalom courses, performed in an interleaved order. Based on their performance on this test, the participants were stratified into two approximately equal groups. Participants in the interleaved group skied all courses each day, executed in an interleaved order under the condition that no more than two runs in the same course would occur consecutively. Participants in the blocked group performed all runs on a single course (i.e., course A, B or C) in a given practice session. The order in which the participants performed the course was counterbalanced across participants. After a retention interval of 72 h, the participants returned to complete a retention test that was similar to the pre-test.

The participants completed the experiment in groups of 10–20. Depending on the size of the ski club and academy, groups consisted of participants from a single ski club or academy or were composed of a larger group from several ski clubs and academies. Participants could continue their regular dryland training, but no ski-related training was allowed during the intervention and test period. See Figure 2 for an overview of the study design.

#### 2.3.1 Pre-test

After completing a demographic questionnaire about their age and gender, participants inspected the three slalom courses. First, they completed two warm-up runs: one free skiing and one in a randomly selected course. Then, participants were instructed to complete 12 runs on each of the slalom courses (course A, B, and C) and three straight-gliding runs in the straight-gliding lane. The participants performed the slalom courses in a semi-random order except for the first and last runs, which were straight-gliding runs. In accord with previous studies, the semi-randomization ensured that no more than two consecutive runs were conducted on the same course. Testing participants in a random rather than a blocked order was done because this procedure has yielded the most notable differences between blocked and interleaved groups. This testing also simulates the competitive environment of alpine skiing, where courses constantly change from one race to another. The participants were instructed to ski the courses as fast as possible, but they did not receive any feedback on their performance from the timing system this day.

After the pre-test, participants attended a workshop where we introduced the concept of “pumping to increase velocity” and the physical principles underlying the effect. The workshop lasted for 30 min and included video materials and empirical evidence from alpine skiing to give a conceptual understanding of the skill. At the end of the workshop, we informed the participants that the goal of the training intervention was to explore pumping motion strategies to maximize the effect of pumping to increase velocity during the three practice sessions. Furthermore, we told them that they did not have a coach for these sessions but should use the feedback system that provided objective feedback to evaluate their performance.

#### 2.3.2 Acquisition

After the pre-test, the participants were quasi-randomly assigned to interleaved or blocked groups based on their pre-test scores. Specifically, for each participant, the best run of the pre-test in each of the three courses was extracted and divided by the average of the straight gliding runs from the pre-test. The participants were then ranked from fastest to slowest and paired in ascending order. Finally, each consecutive pair from this list were shuffled into an interleaved or blocked group.

Participants executed 15 runs each training day: 12 runs on the three courses and three straight-gliding runs in the straight-gliding lane. For the 12 runs executed in the courses, participants in the interleaved group skied all courses each day. The execution of these was randomized in an interleaved order, ensuring that no more than two runs on the same course would occur consecutively. Participants in the blocked group performed all runs on one course (course A, B, or C) on a given day of practice. The order in which the course was performed was counterbalanced across participants.

After each run, all participants received performance feedback from a display at the finish that showed the difference between the actual run and their straight-gliding time (in seconds). Participants were instructed to use the difference between the straight gliding time and the actual run time to evaluate their current performance and try to reach or beat this straight gliding time when skiing in courses A, B, and C using the pumping motion to increase their speed.

#### 2.3.3 Retention

Seventy-two hours after the last practice session, participants returned to complete the retention test, consisting of 12 runs [three runs in each of the three slalom courses (A, B, and C) and three straight-gliding runs]. As in the pre-test, every participant started and ended the testing session with a straight-gliding run. Except for these two straight-gliding runs, the remaining runs were scheduled in a semi-random order to avoid more than two runs being skied consecutively in the same course. Participants were again instructed to ski as fast as possible but did not receive any feedback on their performance during the post-test.

### 2.4 Data processing

#### 2.4.1 Snow condition

To assess the snow conditions, a modified version of the online questionnaire on perceived piste properties, as proposed by Wolfsperger et al. (2015) was employed. Specifically, the participants were asked to judge three characteristics of the snow conditions on the courses each day. The “homogeneity of the course” was assessed on a scale ranging from −3 to 3, where three indicated complete homogeneity of the snow conditions across and within courses, whereas −3 indicated very different conditions. The “mechanical resistance of the snow” was rated on a scale ranging from (−3 to +3), where −3 indicated hardness and +3 indicated softness. Finally, the participants indicated the “grippiness of the snow” on a scale ranging from −3 to +3, where −3 corresponded to grippy and +3 indicated slick/icy. The participants rated these characteristics in the upper and lower part of the course separately. Means and standard deviations of the participants’ responses across courses and upper/lower parts were calculated to describe the snow conditions for each day.

#### 2.4.2 Acquisition

Each run in the slalom courses (A, B, and C) was subtracted from the average straight-gliding time a participant had achieved on the respective training day. The rationale for performing this normalization was to describe how much faster or slower a skier was than his/her straight gliding on the respective day. Also, the normalizing of the data allowed us to compare participants’ performance across days despite minor changes in course length or snow conditions. Runs where a participant for some reason did not finish the course (e.g., due to a mistake or straddling a gate) were omitted. Next, the runs were numbered from 1 to 12 and arranged in ascending order. The runs were subsequently batched into three Acquisitions Trial Blocks (1–4, 5–8, and 9–12 were batched into Acquisition Trial Blocks 1,2 and 3, respectively) for each of the three courses, following the convention adopted from previous studies on contextual interference (Shea and Morgan, 1979; Lee and Magill, 1983). Finally, we calculated the average performance within each batch. Acquisition performance was also calculated and included in the results for participants who dropped out from the retention test.

#### 2.4.3 Retention

Retention was calculated as the average time of the runs in each of the three courses, subtracted from the average straight-gliding time a participant recorded that day. Runs where a participant did not finish the course (e.g., due to a mistake, or straddling a gate) were excluded. This calculation was used to normalize the performance across different days and groups to assess a participant’s performance even if the course length or condition was slightly different.

### 2.5 Statistical analysis

Because participants took part in the experiments as groups of 10–20 from ski clubs and academies during different weeks, external conditions such as snow and the social environment were subject to change. Therefore, we used linear mixed-effect regression models to account for this variation in our models. In addition, linear mixed regression models allowed us to include available information from participants with missing data points in any of the three courses.

To analyze the data, we used linear mixed-effect regression models. To analyze whether the blocked practice group outperformed the interleaved practice group during acquisition, we used a linear mixed-effect regression model to predict performance with Acquisition trial block (1, 2, 3) and Course (A, B, C) as within-subjects factors and group (interleaved, blocked) as the between-subjects factor. To analyze whether the interleaved practice group outperformed the blocked practice group on retention, we predicted retention performance with the main effect and interaction of group (interleaved, blocked) as a between-subjects factor, Course (A, B, C) as the within-subjects factor, and pre-test performance as a covariate. We included a random intercept for both Bib and Academy in both models to account for dependency structure in the data. The models were fitted using the lme4 (Bates et al., 2015) package in R (R Core Team, 2021). *p*-values were obtained from the lmerTest package (Kuznetsova et al., 2017) using the Satterthwaite degrees of freedom method, which yields the most acceptable Type-1 error rate for small sample sizes (Luke, 2017). ANOVA outputs from both these models were reported to ease interpretation and to be consistent with previous literature on the contextual interference-effect. To this end, estimated marginal means were derived from the emmeans package (Lenth, 2021) and the Satterthwaite degrees of freedom. Due to the lack of consensus on calculating standardized effect sizes for linear mixed-effect regression models, we followed the recommendation to report raw effect sizes with a 95% confidence interval (Lohse et al., 2020). We performed visual inspections of the residual plots to assess the uniformity of variance.

We registered the analysis plan after the first round of data collection (https://osf.io/xqte2/). *p*-values < 0.05 were considered statistically significant for the entire study.

## 3 Results

### 3.1 Descriptive data

Descriptive statistics for the participants’ evaluation of the snow conditions each day are presented in Table 1.

**TABLE 1:** The table shows the average rating of the three snow characteristics and the standard deviation in parenthesis for each day. The “*homogeneity of the course*” was assessed on a scale ranging from −3 to 3, where three indicated complete homogeneity of the snow conditions across and within courses whereas -3 indicated very inhomogeneous conditions. The “*mechanical resistance of the snow*” was rated on a scale ranging from (−3 to +3), where −3 indicated hardness and +3 indicated softness. Finally, the participants indicated the “*grippiness of the snow*” on a scale ranging from −3 to +3, where −3 corresponded to grippy and +3 indicated slippery. The participants rated these characteristics in the upper and lower part of the course separately. The mean and standard deviation represented in the table are these two sections’ averages.

Snow conditions					
Snow characteristic	Pre-test	Acquisition day 1	Acquisition day 2	Acquisition day 3	Retention
Grippiness	−1.12 (1.4)	−0.86 (1.3)	−0.89 (1.4)	−0.78 (1.6)	−0.46 (1.8)
Iciness	−1.29 (1.3)	−1.56 (1.2)	−1.39 (1.3)	−1.78 (1.2)	−1.16 (1.6)
The homogeneity of the slope conditions	0.73 (1.8)	0.44 (2.1)	0.39 (1.8)	0.91 (1.8)	1.40 (1.7)

### 3.2 Acquisition

**FIGURE 3:**
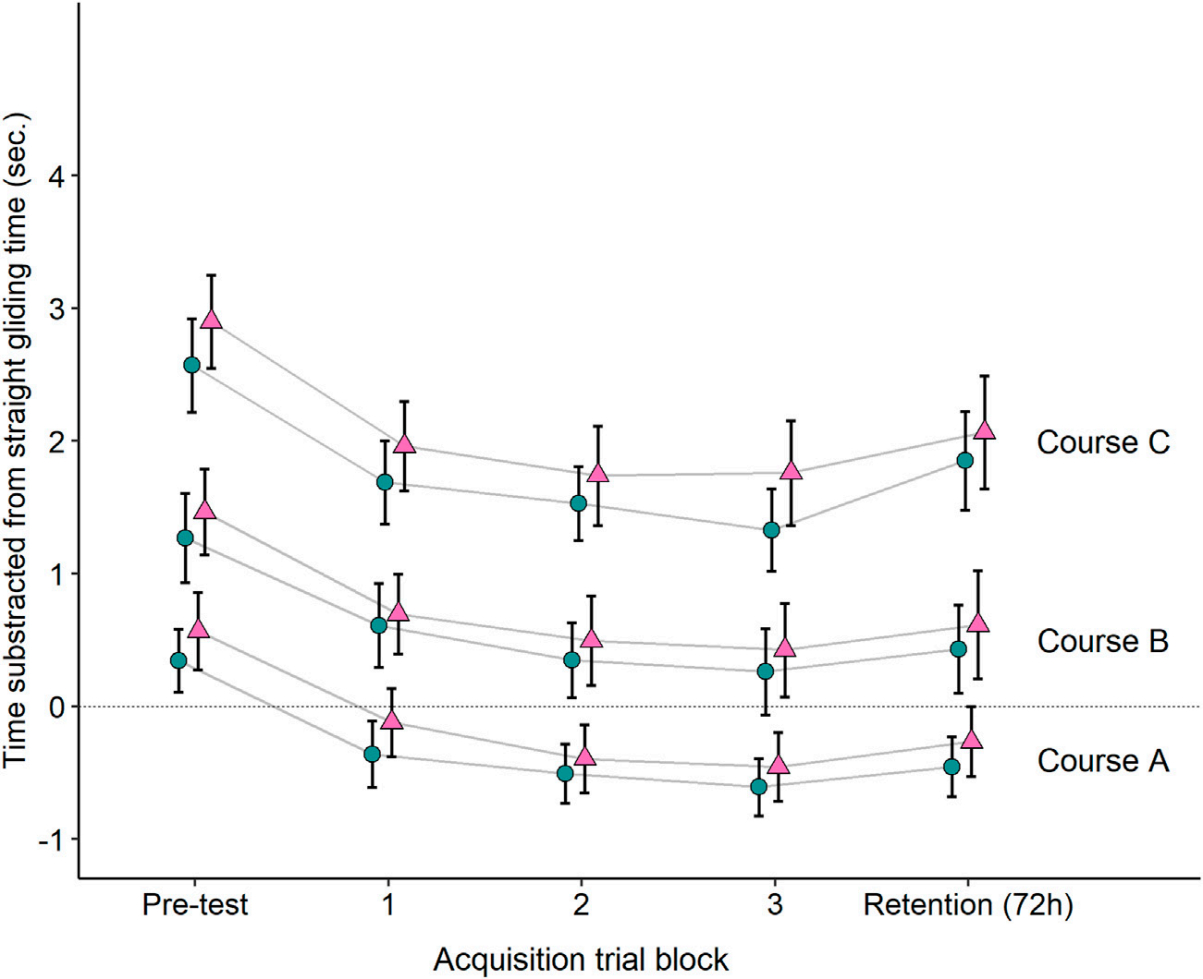
Sample means and 95% confidence intervals for the blocked (red triangle) and the interleaved (blue circle) in courses A, B and C. Performance was computed by calculating the average time the participant achieved for each course and subtracting it from the mean of his or her straight-gliding time on that day. A lower score, therefore, indicates a better performance. The dashed black line depicts when the performance was equal to the straight-gliding performance. To analyze the acquisition, the runs were numbered in the three courses from 1 to 12. We then placed 1–4 into Acquisition trial block 1, 5–8 into Acquisition trial block 2 and 9–12 into Acquisition trial block 3. Please note that not all skiers completed all the training sessions.

Figure 3 shows the acquisition data for the interleaved and blocked groups. The linear mixed-effect regression model revealed a main effect of Time, [F (2, 500.21) = 48.89, *p* < 0.001]. Participants improved their performance from the first [M = 0.70; 95% CI (0.18, 1.23)] to the second [M = 0.49; 95% CI (−0.03, 1.02)] and third [M = 0.37; 95% CI (−0.16, 0.89)] acquisition trial blocks. The model also revealed a main effect of Course, [F (2, 501.94) = 1792.84, *p* < 0.001]. Participants tended to ski course A [M = −0.45; 95% CI (−0.98, 0.08)] in a shorter time than course B [M = 0.42; 95% CI (−0.11, 0.94)] and course C [M = 1.59; 95% CI (1.07, 2.12)]. However, no main effect of Group was observed, [F (1, 61.90) = 0.49, *p* = 0.488]. Averaged across Acquisition trial block and Course, the performance of the blocked group [M = 0.50; 95% CI (−0.13, 1.13)] was only 0.11 s slower than the interleaved group [M = 0.53; 95% CI (−0.10, 1.16)]. No higher-order interactions involving the Group variable were found: Acquisition trial block x Group, [F (2, 500.2) = 2.11, *p* = 0.122]; Acquisition trial block x Group x Course, [F (4, 499.46) = 0.76, *p* = 0.553].

### 3.3 Retention

Figure 3 shows the acquisition data for the interleaved and blocked groups. The linear mixed-effect regression predicting retention revealed a main effect of Course, [F (2, 117.22) = 12.21, *p* < 0.001], when controlling for pre-test performance. This main effect revealed that the participants tended to ski the courses with the shortest offsets (Course A) faster than both other courses, which had larger offsets (Courses B and C). However, no main effect of Group was observed, [F (1, 72.82) = 0.33, *p* = 0.570], when controlling for pre-test performance. The magnitude of difference was only 0.11 s between the interleaved [M = 0.46; 95% CI (−0.06, 0.99)] and the blocked group [M = 0.58; 95% CI (0.04, 1.11)], adjusting for the magnitude of the same difference between the groups at pre-test. No higher-order interactions involving the Group variable were found: Group x Course, [F (2, 116.65) = 0.48, *p* = 0.618]; Pre x Group x Course, [F (2, 101.19) = 1.13, *p* = 0.327]. Hence, our data did not provide evidence for statistical significant group differences in retention in any courses, even when controlling for pre-test performance.

## 4 Discussion

The present study is one of few studies that have addressed the contextual interference effect with skilled learners in a complex task. To test the contextual interference effect, we set three different slalom courses and arranged the trials in these courses differently for the two groups. The participants in the interleaved group practiced each of the three slalom courses each training day in an interleaved order, whereas the blocked group trained the three courses on separate days in a blocked format. We hypothesized that interleaved practice would suppress performance during skill acquisition compared to blocked practice and that interleaved practice would improve retention performance. Contrary to our hypothesis, the data showed that interleaved practice did not significantly suppress acquisition performance or improve retention performance. In other words, our study did not reveal the contextual interference pattern previously reported in the motor learning literature. Both groups improved substantially during the intervention, but no reliable difference between the groups was observed.

The result of this study conflicts with the substantial body of research that has found the contextual interference effect to be present in a wide range of different laboratory tasks (e.g., Shea and Morgan, 1979; Magill and Hall, 1990; Simon and Bjork, 2001). A simple explanation for the divergence of this result from most scientific findings is that the skill level of our participants was considerably higher, and the skill type was more complex than in most previous studies on the contextual interference effect in motion tasks. In accordance with this view, a study with comparable characteristics to our study that addressed the contextual interference effect with skilled performers did not find that interleaved practice enhanced retention compared to blocked practice (Buszard et al., 2017). These researchers only found an advantage of interleaved over blocked practice in transferring the skills to competition performance. Therefore, gains in retention may not occur for skilled performers learning complex tasks following interleaved practice. However, this explanation conflicts with the findings of Hall et al. (1994), who found that interleaved practice improved retention for skilled batters compared to blocked practice. It is critical to note that this study manipulated the contextual interference between variations of the same skill (i.e., learning to bat in response to three different pitches) instead of manipulating it between skills like Buszard et al. (2017). Therefore, it could be that improvement in retention following interleaved practice will only appear for skilled performers if the manipulation occurs between variations of the same skill. However, this interpretation conflicts with our data pattern because we also performed the manipulation between variations of the same skill.

An alternative perspective on our results pertains to the characteristics of the skill used in our study. Alpine skiing is a continuous skill that operates heavily on feedback control mechanisms (for example, reacting to incoming visual and tactile information) entering the system to regulate motion while skiing through a slalom course (Diedrichsen and Kornysheva, 2015). Because of the long duration of a run in skiing (>40 s), more sensory information is potentially available and harnessed during the execution of the skill compared to the execution of discrete skills, which last for a much shorter time (Lee and Genovese, 1988, Lee and Genovese, 1989). The continuous characteristic and long run duration let participants evaluate their performance during execution and allow for rapid adjustments early and repeatedly during a run to sustain high-performance levels. This type of skill contrasts with discrete skills that have a shorter duration and, therefore, would not allow such adjustments during the run based on sensory feedback acquired during execution. Following this reasoning, with continuous skills, any potential interference from switching between courses may not be as influential because the learner may have sufficient time to adjust their performance during a trial. Although this explanation may contradict previous research that has observed the contextual interference effect with continuous skills, such as bimanual learning task (Tsutsui et al., 1998; Pauwels et al., 2014) and snowboarding turns (Smith, 2002), to our knowledge, no study has examined continuous tasks with skilled performers. From a challenge-point framework (Guadagnoli and Lee, 2004) point of view, it could be argued that the extensive stream of sensory signals that performers receive during skill execution is more interpretable and informative for skilled than for novice learners. Hence, skilled participants might better understand the feedback they get during a trial, allowing them to make necessary adjustments to accommodate optimal performance more effectively than novice learners. In effect, interleaved practice will impose different effects on performers with different skill levels that learn continuous tasks: for beginners, it will create favorable conditions for learning, but for skilled learners, it might not create stimuli sufficiently different from regular practice. This account may explain why Smith (2002) provided evidence for improved retention for beginners learning snowboarding turns, whereas our data did not support such a relationship. Therefore, future studies should further address the contextual interference effect for continuous tasks with skilled performers and primarily address the role the level of stimulus has on the contextual interference effect.

Another reason the contextual interference effect did not appear in the present study could be that the difference between the three slalom courses was not sufficiently large to probe the contextual interference effect. Although contextual interference research does not precisely detail the type or magnitude of contrast between tasks necessary to produce the contextual interference effect (Ramezanzade et al., 2022), this scenario is a potential limitation that needs consideration when evaluating this study. We maximized the offset differences that were within reason for the selected gate distance. Specifically, we sought to find a balance between the need for maximal differences while allowing for active pumping within the space constraints of the skiing hall. Previous research has also shown that changes in gate offset (of the magnitude we used in our study) significantly impact the skiers’ technique and tactics (Spörri et al., 2012; Gilgien et al., 2015; Gilgien et al., 2021), providing evidence that the gate offset variation we chose adequately created contextual interference. However, it may be that the stimulus effect of the three courses changed during the intervention, such that a participant who got better at pumping needed to execute this skill in each course with a higher frequency or a different line selection than before the intervention. Consequently, the courses may have changed their impact on skiers’ technique and tactics because the participants improved during the intervention. If so, the blocked group may also have experienced some interference each day, which may explain why we did not observe a contextual interference effect.

It is also conceivable that the long time that elapses between runs in typical alpine skiing sessions and in our study may have impacted the results. Participants used approximately 8 min between trials to ride the lift to the top and prepare themselves for a new run. This inter-trial interval is significantly longer than the inter-trial interval used in laboratory tasks, where discrete skills were tested (Barreiros et al., 2007). It may be that the time between trials was so long that athletes forgot and lost connection between trials, which is suggested to be one of the mechanisms causing the contextual interference effect (Lee and Magill, 1983, 1985). This interpretation similarly relates to the new theory of disuse (Bjork and Bjork, 1992). This theory assumes that lengthening the spacing between trials may increase forgetting yet enhance learning by reducing the retrieval strength of memory before every trial. In this view, both the interleaved and the block group may have experienced favorable conditions for skill learning due to the long time between trials. Suppose this explanation hindered any contextual interference effect from coming into place in our study. In that case, the contextual interference effect might never be present in alpine skiing due to the long time required to transport the skiers back to the start of the racetrack and establish the inter-run recovery needed. Alpine ski racing causes acute fatigue, requiring a break to recover between runs to avoid declining performance during the session due to accumulated physical and psychological fatigue (Turnbull et al., 2009; Ferguson, 2010). However, future investigations should examine whether the length of the inter-trial time affects contextual interference in general and especially as related to complex continuous skills.

A further point for discussion is that this study took a replacement approach to perform the training, whereby participants replaced their regular ski training entirely with that offered in the experiment. This strategy allowed us to minimize the number of intervening factors (for example, maturation and stress) during the experiment. By contrast, previous studies on contextual interference with skilled performers have performed the experiment by adding extra practice to the participant’s regular training (Hall et al., 1994; Buszard et al., 2017). Consequently, the experiment often spans multiple weeks. Based on the evidence that distributing practice over an extended period can benefit learning (Lee and Genovese, 1988), the different approaches to examining contextual interference with skilled performers may be one reason for the observed differences between the studies.

Finally, we would like to consider two alternative interpretations for why we did not observe the contextual interference effect. The first reason is that interleaved practice does not alter task difficulty for these complex skills and this group of learners (Farrow and Buszard, 2017). This account posits that contextual interference is missing in this context unless an assessment of learning adopts a transfer criterion for learning. Unfortunately, we could not establish a transfer test for this study due to various constraints (e.g., access to the skiing hall). Therefore, an interleaved practice may improve learning, but the study could not capture this aspect of learning with the chosen design. In line with others (Buszard et al., 2017; Farrow and Buszard, 2017), we recommend that future studies on the contextual interference effect include transfer tests to understand better the contextual interference effect for complex tasks and skilled learners. The other explanation is that the contextual interference effect is usually more reliably observed with tasks with solid visual requirements (Apidogo et al., 2021; Schöllhorn et al., 2022). This account may explain why (Hall et al., 1994) observed improved retention in the batting task that required batters to perceive the type of throw for each trial, whereas Buszard et al. (2017) and we did not observe this relationship.

There are several potential caveats that should be considered when evaluating the findings of this study. First, this was a real-world learning study where groups of skiers from different ski clubs and academies trained together. Although this may have enhanced the ecological validity of the study because it mimics the skiers’ regular training, we cannot eliminate the possibility that interactions between the skiers may have influenced the results. In general, the participants were quite spread around the hill during the experiment, but they could observe each other at the start or from the chairlift. Another point to consider is that, although we did everything to create proper and stable conditions, variation in the courses or conditions is inevitable, even in the indoor skiing hall.

## 5 Conclusion

To conclude, the unique strength of this study was the approach adopted to examine contextual interference in a complex sport with skilled participants. We recruited many skilled participants by creating a training intervention that targeted a specific skill the participants were willing to invest time and effort to improve. In contrast with the substantial body of literature on the contextual interference effect, our data did not provide support for the presence of the contextual interference effect using an interleaved training design. Some explanations for why we did not find support for the contextual interference effect include the skill level of our participants and the continuous and complex characteristics of the task. It is also essential to consider whether the manipulation was sufficiently large to create contextual interference. Even though our data did not support the contextual interference effect, we do not suggest that coaches should de-emphasize the importance of variability and changing courses in their training with alpine skiers. For example, frequently switching between different courses is suggested to be an effective strategy to enhance retention and transfer of skills in this sport because it mimics the competition environment (Farrow and Buszard, 2017). However, the effect may lag in time and not become apparent until several years of practice have been completed. We also provided evidence that interleaved practice did not degrade performance during acquisition. Therefore, coaches can safely employ interleaved practice training in alpine ski racing. Because many factors affect contextual interference in complex tasks with skilled performers, future research should continue studying the contextual interference in naturalistic environments, involving continuous tasks and skilled performers.

## Data Availability

The raw data supporting the conclusion of this article will be made available by the authors, without undue reservation.
