# Supplementary material for: Is there a contextual interference effect for sub-elite alpine ski racers learning complex skills?
Source: Front Bioeng Biotechnol. 2022 Sep 15;10:966041. doi: 10.3389/fbioe.2022.966041 (PMC9521505; doi:10.3389/fbioe.2022.966041)
Supplement: Supplementary file 1 [file Table1.DOCX]

Power calculation

For our power calculation, we used G*Power 3 (Faul et al., 2007). Specifically, we calculated the statistical power achieved by our sample size of 54 for a small and medium effect. For our calculations, we selected Analysis of Covariance (ANCOVA) and calculated power for the main effect (that is, group difference) with one covariate. We performed these calculations with 1 numerator Df because our Group variable had two levels (interleaved and blocked). Here is the protocol for the two power analysis that we calculated:

**Small effect size**

**F tests -** ANCOVA: Fixed effects, main effects and interactions

**Analysis:** Post hoc: Compute achieved power

**Input:** Effect size f = 0.10

α err prob = 0.05

Total sample size = 54

Numerator df = 1

Number of groups = 2

Number of covariates = 1

**Output:** Noncentrality parameter λ = 0.5400000

Critical F = 4.0303926

Denominator df = 51

Power (1-β err prob) = 0.1113654

**Medium effect size**

**F tests -** ANCOVA: Fixed effects, main effects and interactions

**Analysis:** Post hoc: Compute achieved power

**Input:** Effect size f = 0.25

α err prob = 0.05

Total sample size = 54

Numerator df = 1

Number of groups = 2

Number of covariates = 1

**Output:** Noncentrality parameter λ = 3.3750000

Critical F = 4.0303926

Denominator df = 51

Please note that we used linear-mixed effect regression for our analysis of the data, which uses maximum likelihood as its estimation machinery instead of OLS (Long, 2012). Hence, the tests are not identical.

**References**

Faul, F., Erdfelder, E., Lang, A.-G., and Buchner, A. (2007). G*Power 3: A flexible statistical power analysis program for the social, behavioral, and biomedical sciences. *Behavior Research Methods* 39(2)**,** 175-191. doi: 10.3758/BF03193146.

Long, J.D. (2012). *Longitudinal Data Analysis for the Behavioral Sciences Using R.* Los Angeles: SAGE Publications.
